# Effect of Milking Method, Diet, and Temperature on Venom Production in Scorpions

**DOI:** 10.1093/jisesa/iey081

**Published:** 2018-09-04

**Authors:** Saadia Tobassum, Hafiz Muhammad Tahir, Muhammad Tariq Zahid, Qurratulann Afza Gardner, Muhammad Mohsin Ahsan

**Affiliations:** 1 Department of Zoology, Government College University Lahore, Punjab, Pakistan; 2 School of Biological Sciences, University of the Punjab, Quaid-e-Azam Campus, Lahore, Punjab, Pakistan; 3 Department of Zoology, University of Education, DG Khan Campus, Punjab, Pakistan

**Keywords:** scorpions, venom extraction, electrical method, food, temperature

## Abstract

In the present study, two common buthid scorpions, i.e., *Androctonus finitimus* (Pocock, 1897) (Scorpiones: Buthidae) and *Hottentota tamulus* (Fabricus, 1798) (Scorpiones: Buthidae), were maintained in the laboratory for venom recovery. The aim of study was to compare the quantity and quality of venom extracted from scorpions by manual and electrical method. We also recorded the effect of diet and temperature on venom production. Results of our study revealed that electrical method yielded good quality and higher quantity of venom as compared to manual method. The quantity of venom by two studied species differed statistically. We recorded the effect of food on venom production by providing different prey items to the scorpions and found that grasshopper nymphs and adults were the best diet for the scorpions to get maximum yield of venom as compared to other prey types (house crickets, house flies, and moths). Production of venom and activity of scorpions was found to be associated with temperature. During winter season, venom recovery was comparatively low as compared to the hottest part of year; when venom milking and activity of scorpions both were increased.

Scorpion stings cause significant morbidity and mortality in many parts of the world (Ismail 1995, Al Asmari et al. 2014). Scorpion venom is a highly complex mixture of molecules, which can disturb physiological activity of host by envenomization (Ding et al. 2014). Despite of various negative effects, it contains many different biologically active components which are being used for the development of drugs in the pharmaceutical industries (Andreotti et al. 2010, AbdulRahman et al. 2016, Zhang and Zhang 2016). Scorpion venom had been widely useful in traditional medicines and current pharmaceutical research. Therefore, venom milking or venom extraction has been the primary step of such investigatory studies. So, it is crucial to maintain and rear the scorpions in the laboratory for venom milking and conservation. In previous studies, different methods have been used for venom milking of scorpions such as maceration of telson (Ozkan et al. 2006), mechanical stimulation (Oukkache et al. 2013), and electrical stimulation (Diaz-Garcia et al. 2015). The quantity and composition of venom vary greatly with each method of extraction (Oukkache et al. 2013).

Being the oldest venomous species, scorpions had been extensively studied for venom (AbdulRahman et al. 2016). Venom varies in composition from species to species and may differ in potency due to the changes in their toxins or associated with environmental and genetic variations, such as climate and diet (Rodriguez-Ravelo et al. 2013, Pucca et al. 2014). However, understanding the effect of diet and temperature is essential for maintaining the scorpions in the laboratory for venom extraction. Ecological factors, i.e., relative humidity, air, and temperature, affect the activities of scorpions (Bridges et al. 1997, Ahsan and Tahir 2016).

For centuries, scorpion venom had been used in traditional medicine in Africa, Cuba, India, and China (Gomes et al. 2010). Scorpions and their venoms are very effective for the treatment of various diseases. They have analgesic, antiepileptic, anticancer, anti-inflammatory, antimicrobial, and hemolytic activities (Yu et al. 1992, Almaaytah and Albalas 2014, Harrison et al. 2016). In addition to this, in China dried bodies of scorpions have been used as analgesic and anti-epilepsy agent since Song Dynasty (A.D. 960–1279) (Shao et al. 2007). Scorpions and their venom are imperative for medical research; therefore, scorpion rearing is important and difficult task for venom extraction.

Scorpion venom can be used for different purposes such as antivenom development, disease diagnosis, basic research, and most importantly for drug development. To get considerable amount of venom, it is important to rear and maintain the scorpions in the laboratory. For this, we should be aware of different factors such as temperature, humidity, and food preferences which affect venom production. Keeping in view above facts present study was conducted. The aim of study was to compare the quantity and quality of venom extracted by manual and electrical method and determine the effect of diet and temperature on venom production.

## Methods

### Collection of Scorpions and Their Maintenance

**Fig. 1.**
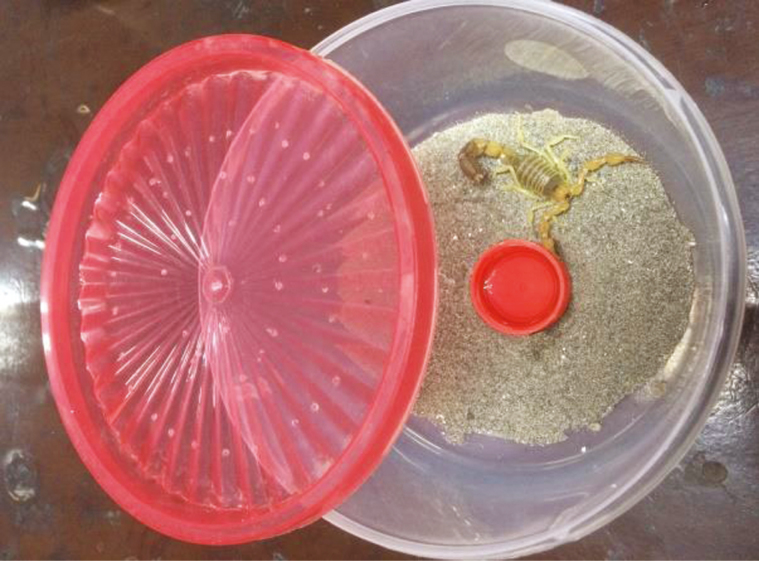
Plastic box used to keep collected scorpions. Each box was layered with sand and covered with a lid containing holes for proper aeration.

The study was conducted from February 2017 to January 2018. *Hottentota tamulus* (*n* = 60) and *Androctonus finitimus* (*n* = 60) scorpions were collected from sandy areas of Mianwali and Sargodha Districts of Punjab, Pakistan (i.e., Mianwali: Chak # 16/DB, 32°38′77″N, 71°31′69″E; Bhagtanwala: Chak # 34/SB, 31°55′59″N, 72°54′22″E; Chak # 76/SB, 32°06′74″N, 72°52′38″E; Chak # 24/SB, 32°02′07″N, 72°55′48″E). Scorpions are nocturnal creature, so they were collected at night with the help of portable battery equipped with UV light (SOGO-JPN-139, Japan). They were brought to the laboratory in the plastic jars (15 × 7 × 7 cm) (L × W × H) and were placed in the fairly small transparent plastic boxes (17 × 9 × 9 cm) (L × W × H) containing 2–3 inches substrate of sand at their bottom. The lid of these boxes was designed in such a way that they provide proper ventilation and temperature (Fig. 1). They were placed away from direct sunlight because they are nocturnal.

### Scorpion Rearing

**Fig. 2.**
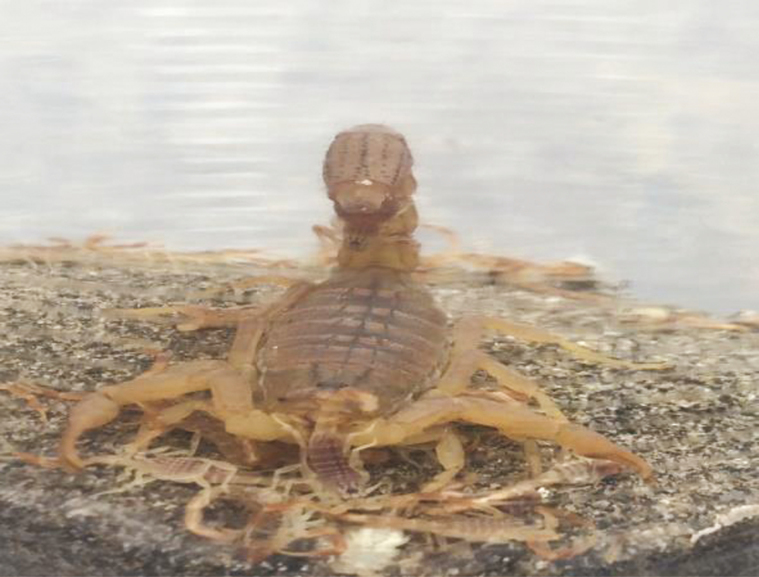
Young ones resided on the back of female scorpion.

During rearing, females bearing juveniles were separated from the field collection and placed in separate 1,000-ml round flask (Fig. 2). Their young one resided on the back of mother. Baby scorpions were separated from the mother and maintained in the laboratory under control conditions. Only the healthy adult male scorpions (*n* = 60) of both species (*H. tamulus* and *A. finitimus*) ranging from 7.5 to 8 cm were included in the experiment. Male scorpions were preferred as they produce significantly higher yield of venom than female scorpions according to our previous experience.

### Food and Water for Scorpions

**Fig. 3.**
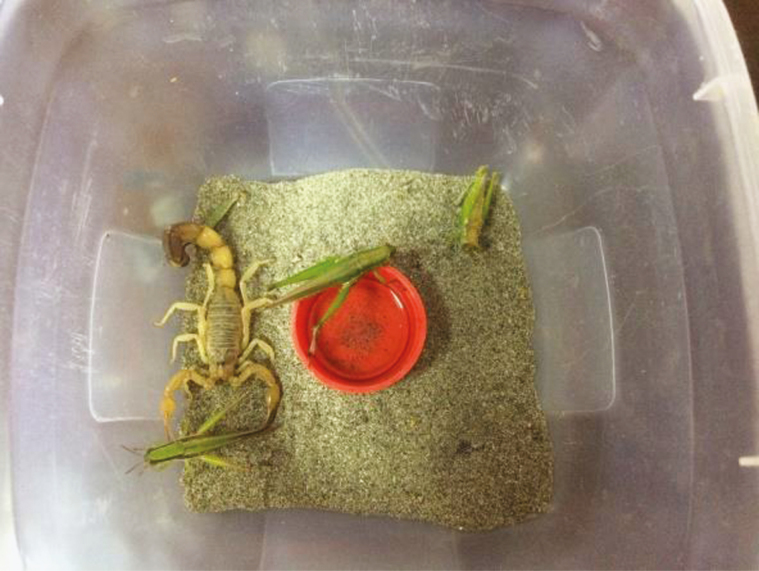
Scorpion feeding on live grasshoppers.

In summer, water was periodically sprayed with a wash bottle at the substrate which preserves high humidity, whereas in winter, a shallow-bottomed water dish was placed in the center of each box (Fig. 1). Live insects such as grasshopper nymphs, grasshopper adults, house crickets, house flies, and moths were offered to the scorpions as a food once in a week (Fig. 3).

### Cleanliness of Scorpion Boxes

Scorpions not made much mess but their boxes were regularly cleaned. For this, food remnants were removed daily and sand was changed after every 1 mo. Scorpions do not feed properly if they are placed in unsuitable conditions. So they were maintained at 25–40°C (temperature), 50–60% (humidity), and 12:12 (L:D) h cycle.

### Manual Extraction of Venom

**Fig. 4.**
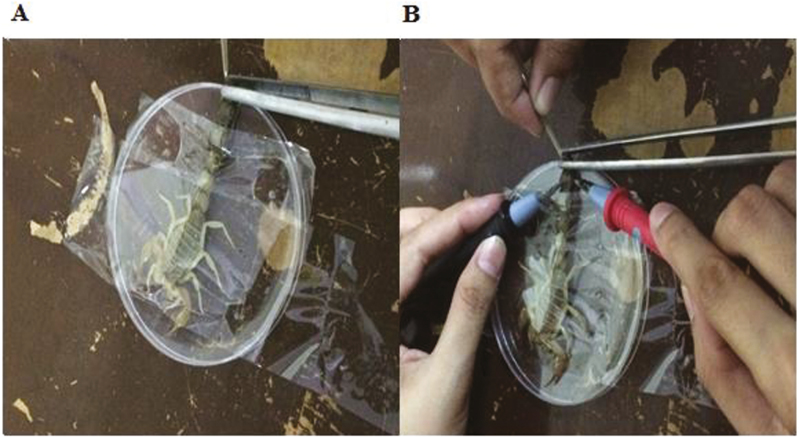
Methods of venom milking in scorpions: (A) manual method and (B) electrical method.

Venom of *A. finitimus* and *H. tamulus* was milked manually by method described by Louis (1976). In this method, scorpion was placed on a petriplate with the help of tape and their stinger was inserted in the tip of graduated capillary tube (Fig. 4A). Then abdomen of scorpions was manually stimulated which release venom. Indeed, many researchers revealed that manually milked venom is the first type of venom denominated as prevenom, whereas venom extracted electrically correspond to the second type (Oukkach et al. 2013).

### Electrical Extraction of Venom

Another method used for extraction of venom was electrical stimulation of telson. For this, the method described by Yaqoob et al. (2016) was followed. For extraction of venom, scorpion was placed on a petriplate with sticky tape. With the help of pointed electrode, electric current (25 V) (Yaqoob et al. 2016) was applied at the base of telson for 5 s to shock the scorpions until the venom was released (Fig. 4B). For this purpose, we immersed scorpion body in 10% NaCl solution for better conductance of electric current. The venom was collected in a graduated capillary tube and mixed with distilled water in 1:5 ratio (venom:distilled water). Diluted venom was centrifuged at 14,000 rpm for 10 min in a centrifuge (MPW-352R, Germany). The supernatant was stored at −20°C for further use.

### Matrix-Assisted Laser Desorption Ionization Time of Flight Mass Spectrometry

Both types of venom (manual and electrical) were further analyzed by MALDI-TOF MS (matrix-assisted laser desorption ionization time of flight mass spectrometry) using MALDI-TOF/TOF Autoflex III (Bruker Daltonik GmbH, Germany). Dried droplet method was used for the preparation of sample. Two microliters of sample was mixed with 8 μl of α-cyanohydroxycinnamic acid solution (10–12 mg/ml in 0.1% trifluoroacetic acid (TFA) in acetonitrile and 0.1% TFA in water, with a ratio of 1:2). One microliter of sample was spotted on MALDI plate (MTP Anchor chip var. 384), dried, and analyzed in linear positive mode as detailed in Gardner et al. (2013).

### Effect of Food on Venom Production

For recording the effect of diet on scorpion venom in the laboratory, common prey items were offered to the scorpions, i.e., grasshopper nymphs and adults, *Acrida conica* (Fabricius 1781); house crickets, *Acheta domestica* (Linnaeus 1758); moth, *Manduca quinquesmaculata* (Haworth 1803); and house flies, *Musca domestica* (Linnaeus 1758). For collection of prey suction device (Siemens VK20COI, Germany) and sweep nets were used (Butt and Tahir 2010). Foraging activities of *H. tamulus* and *A. finitimus* both in the field and laboratory were recorded by Ahsan and Tahir (2016) which revealed that these species of scorpions consumed high number of these prey items, so we studied the effect of above-mentioned prey types on venom production. Scorpions were acclimatized for 1 mo under laboratory conditions before using in the experiment. Scorpions of both species were divided into five groups separately (*n* = 8 in each group). First group was provided with grasshopper nymphs. Adult grasshoppers were offered to the second group. House crickets and moths were provided to the third and fourth experimental groups, respectively. However, fifth group was provided with house flies. It was ensured that total biomass of the prey items offered to each group remained the same. Venom was extracted from individuals of each group after starvation of 7 d and quantity of venom in µl was recorded. To determine the volume of venom, extracted venom was collected in a graduated capillary tube. The experiment was replicated thrice.

### Effect of Temperature on Venom Production

To study the effect of temperature on venom production, eight scorpions of each species were reared whole year in the laboratory. During winter season, scorpions were maintained at 15–20°C because in the field they survive at that temperature and in the summer season they were maintained at 30–40°C according to the field temperature. Venom was extracted from the scorpions at every month and venom quantity (µl) was recorded by a graduated capillary tube.

### Statistical Analyses

Normality of data was assessed by Shapiro–Wilk test before applying statistics. Two-sample *t*-test was applied on the raw data for the comparison of venom quantity extracted from two scorpion species. One-way analysis of variance (ANOVA) followed by Tukey’s test was applied on the data to compare the quantities of venom extracted from different groups of scorpions reared on different food types (feeding test). Pearson correlation was used to find the relationship of venom production with temperature. All statistical analyses were performed using SPSS 15. Results were considered significant if *P* value was less than 0.05.

## Results

**Fig. 5.**
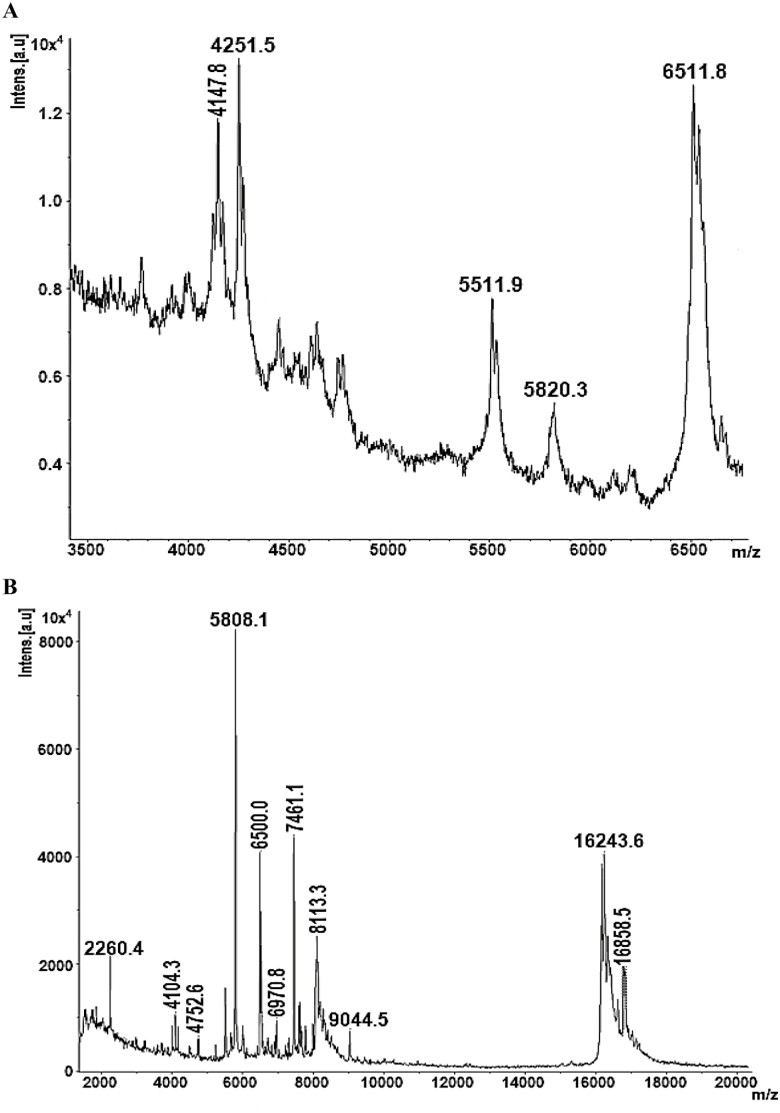
MALDI-TOF MS of venom extracted by manual and electrical method in *A. finitimus*: (A) manual method and (B) electrical method.

**Fig. 6.**
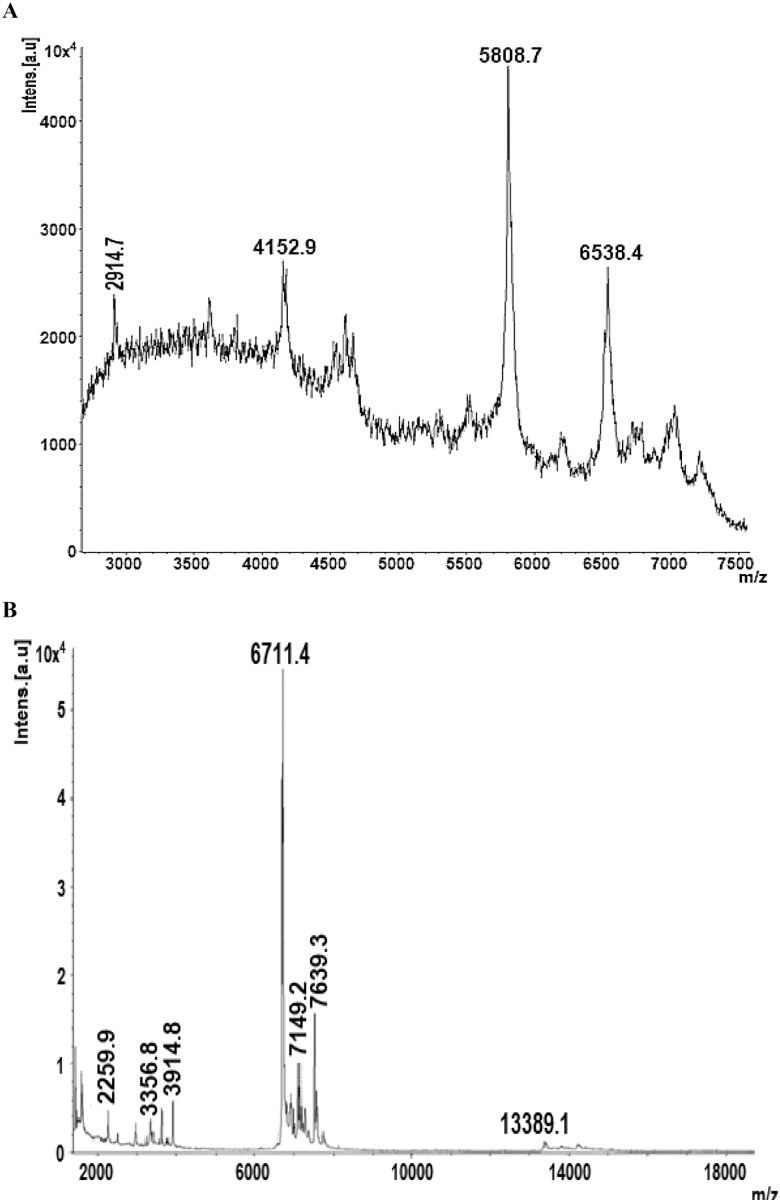
MALDI-TOF MS of venom extracted by manual and electrical method in *H. tamulus*: (A) manual method and (B) electrical method.

In our study, we compared the quality of venom by its color in both electrical and manual method of extraction (Fig. 4). Our results indicated that venom extracted by electrical method is white and remained white after milking in both *A. finitimus* and *H. tamulus* scorpions. To contrary, color of manually extracted venom was changed rapidly after milking in both scorpion species. But on the basis of color, we cannot differentiate these two types of venoms, so we analyzed the venoms by mass spectrometry. In MALDI-TOF MS, we noted that manually extracted venom of *A. finitimus* showed 5 reproducible peaks, whereas electrically extracted venom of same species produced 12 peaks. In manually extracted venom of *A. finitimus*, most of the peaks range from 4,000 to 7,000 Da. However, in electrically extracted venom the range of peak was 2,000–17,000 Da (Fig. 5A and B). Manually and electrically extracted venom of *H. tamulus* produced 4 and 8 reproducible peaks, respectively, by MALDI analysis. In manually extracted venom of this species, most of the peaks range from 2,000 to 7,000 Da, whereas in electrically extracted venom the range of peak was 2,000–14,000 Da (Fig. 6A and B). Components greater than 7,000 Da were not seen in manually extracted venom of both species but were present in electrically extracted venom (that is why scale beyond 7,000 not shown in manual ones so that we could obtain a much magnified peaks whatever were obtained).

**Fig. 7.**
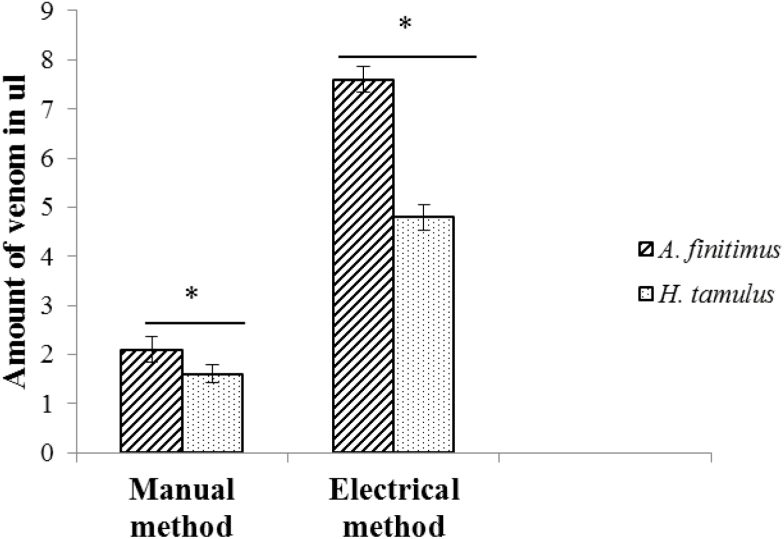
Comparison of venom production by manual and electrical method in *A. finitimus* and *H. tamulus*. * indicate that venom milking was significantly high (*P* < 0.01).

**Fig. 8.**
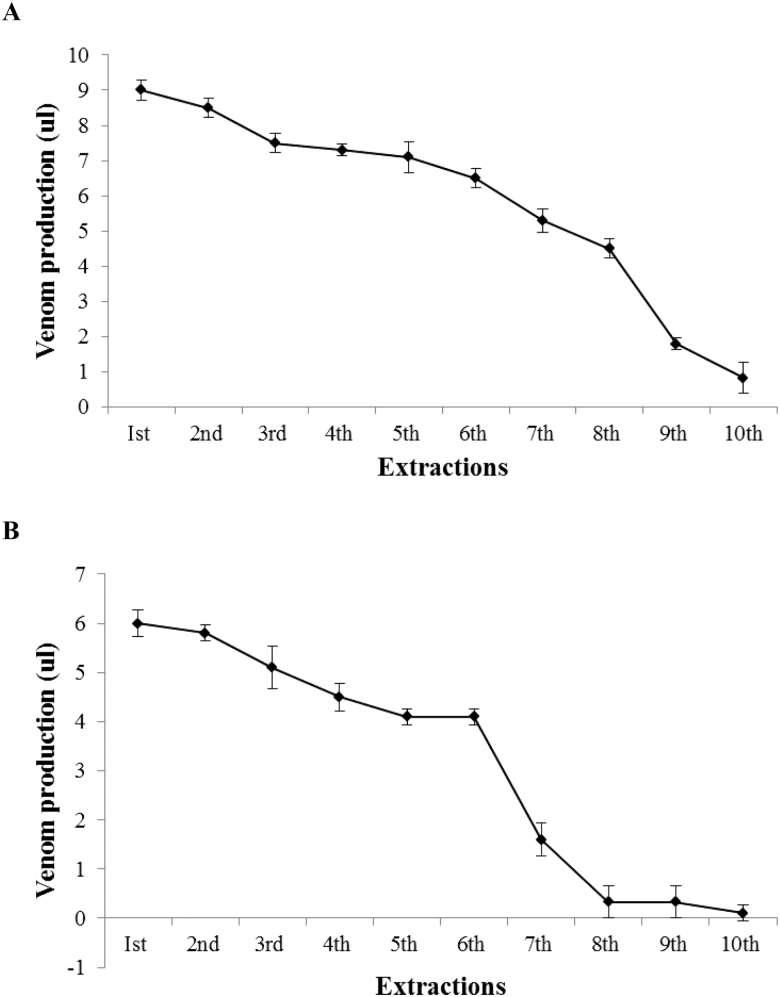
Decrease in venom production after consecutive extractions (A) *A. finitimus*, (B) *H. tamulus*.

In manual method, we recovered 2.1 ± 0.26 (µl) venom from *A. finitimus* and 1.6 ± 0.18 (µl) venom from *H. tamulus*, whereas in electrical method we got 7.6 ± 0.26 (µl) and 4.8 ± 0.26 (µl) venom from *A. finitimus* and *H. tamulus*, respectively. The yield of venom extracted by electrical method was significantly higher than the venom yield by manual method in both species (*t* = −67.36; df = 8; *P* < 0.01 for *A. finitimus* and *t* = −39.19; df = 8; *P* < 0.01 for *H. tamulus*). Our results have shown that in both methods of extraction quantity of venom was statistically higher in *A. finitimus* than *H. tamulus* (*t* = 34.29; df = 8; *P* < 0.01 for electrical method and *t* = 6.124; df = 8; *P* < 0.01 for manual method, Fig. 7). In our study, first time we recovered 9 µl venom from *A. finitimus* which was gradually decreased to 4.5 µl at eighth extraction (Fig. 8A). In *H. tamulus*, 6 µl venom was recovered at first extraction which was gradually decreased to 1.6 µl at seventh extraction (Fig. 8B) (time between two successive extractions was 15 d). We observed that *H. tamulus* did not yield venom after seven extractions.

**Fig. 9.**
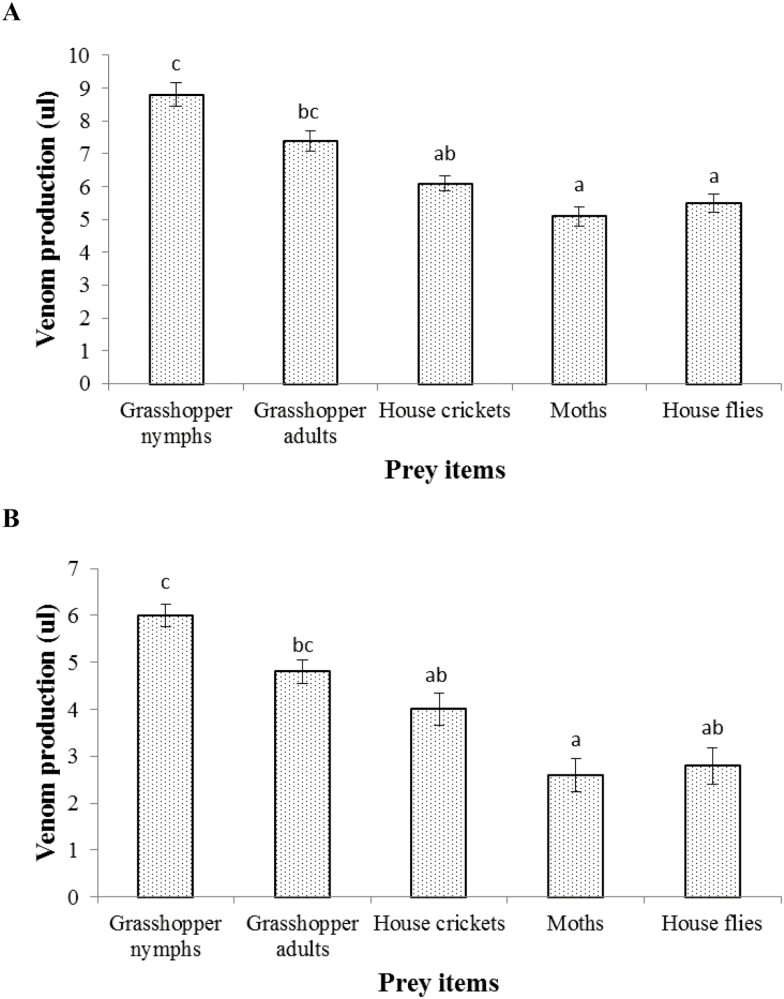
Amount of venom (µl) extracted from experimental groups after giving specific food to each group in *A. finitimus* (A) and *H. tamulus* (B). In the figure bar having different alphabets shows significant difference.

In the present study, we offered different prey items to the scorpions and compared the quantity of venom in both species (Fig. 9A and B). We recorded that nymphs and adults of grasshoppers were the best food for the scorpions (*n* = 8) to get good quality and maximum yield of venom in both species. Venom quantity was also high when house crickets were provided to the scorpions (*n* = 8) but it was less than the grasshopper prey. Recovery of venom was very poor when house flies and moths were offered to the scorpions (*n* = 8). In *A. finitimus*, we get 8.8 ± 0.36 (µl), 7.4 ± 0.31 (µl), 6.1 ± 0.24 (µl), 5.1 ± 0.29 (µl), and 5.5 ± 0.28 (µl) venom from different groups of scorpions when grasshopper nymphs, grasshopper adults, house crickets, moths, and house flies were offered as a food, respectively, whereas in *H. tamulus*, we get 6.0 ± 0.25 (µl), 4.8 ± 0.26 (µl), 4.0 ± 0.35 (µl), 2.6 ± 0.36 (µl), and 2.8 ± 0.39 (µl) venom, respectively, when above-mentioned prey items were provided as a food. However, results of ANOVA followed by Tukey’s test showed that the scorpions which were fed with grasshopper nymphs yielded significantly higher venom than the other scorpions which were offered with other prey items (*F*_4,10_ = 15.40; *P* < 0.05 for *A. finitimus*, Fig. 9A and *F*_4,10_ = 11.02; *P* < 0.05 for *H. tamulus*, Fig. 9B).

**Fig. 10.**
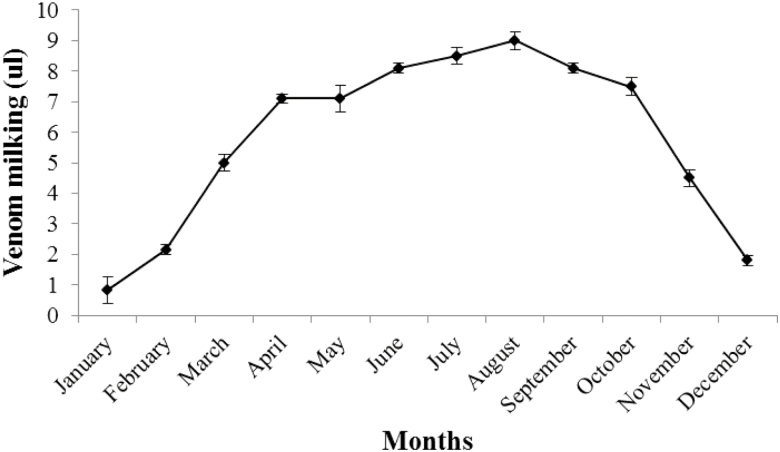
Effect of temperature of different months on venom production of *A. finitimus* by electrical stimulation.

**Fig. 11.**
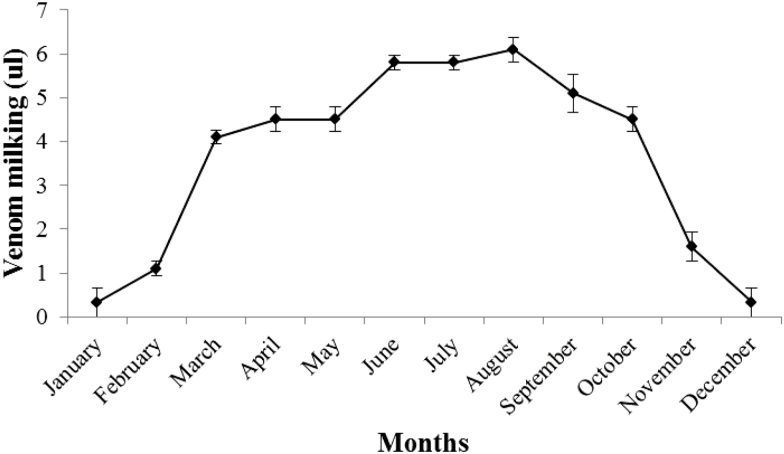
Effect of temperature of different months on venom production of *H. tamulus* by electrical stimulation.

Naturally, scorpions are more active in the summer and their activity decreased in the winter season as temperature drop. In the present study, we maintained the scorpions (*n* = 8) for venom extraction whole year in the laboratory to ensure that behavior of scorpions with respect to venom production is similar in the laboratory conditions as they behave in the field and venom production according to seasonal variation was observed because scorpion venom has developed much attraction for pharmaceutical purposes because of its toxic peptides. We recorded that venom production and activity of scorpions was associated with temperature. During winter season (December, January, February) when the temperature was low, venom production was decreased from scorpions of both species and activity of scorpions was also decreased during winter season. Whereas during hottest part of year (June, July, August) venom production of both species was increased (Figs. 10 and 11). Results of Pearson correlation have shown that scorpion activity and venom milking was better at higher temperature than low temperature. Significant, positive correlation was found between venom production and temperature (*r*^2^ = 0.957 for *A. finitimus* and *r*^2^ = 0.955 for *H. tamulus*).

## Discussion

In the present study, we observed the effect of milking method, diet, and temperature on venom production in most common scorpions, i.e., *A. finitimus* and *H. tamulus* of Punjab, Pakistan. In our study, we determined the quality and quantity of venom extracted by both manual and electrical method. We revealed that electrical method yielded good quality and higher quantity of venom as compared to manual method. Primary characteristic of venom is its quality which is determined by its color. Many researchers had reported similar results (Latifi and Tobatabai 1979, Oukkach et al. 2013). Oukkach et al. (2013) have reported that color of electrically extracted venom remained white after milking, whereas color of venom extracted by manual method rapidly turned to blue after milking in *Androctonus mauretanicus* and *Buthus occitanus* scorpions. Inceoglu et al. (2003) have observed that initial stimulation of scorpions release small amount of transparent venom named as prevenom, whereas further electrical stimulation release dense and cloudy secretion which is white in color. Latifi and Tobatabai (1979) have reported that average amount of venom extracted by electrical method was 0.3 mg per scorpion (*A. crassicauda*), whereas 0.5 mg venom was extracted by maceration of telson.

Manually extracted venom (prevenom) contains several peptides and salts that change the ion channels and produce significant toxicity and pain because of the massive depolarization (Oukkach et al. 2013). In present study, we found that manually milked venom contains low concentration of peptide toxins as compared to the electrically milked venom which contains higher concentration of peptides, our results confirms the observations of many researchers published by Inceoglu et al. (2003) and Oukkach et al. (2013). In MALDI-TOF MS, we observed that manually extracted venom of *A. finitimus* and *H. tamulus* showed 5 and 4 reproducible peaks, whereas electrically extracted venom of both species contain 12 and 8 major peaks, respectively. Electrically induced venom of both species showed a higher number of peaks than manually milked venom, identified by mass spectrometric analysis. This might be due to the fact that electrically milked venom contains a large number of components than manually milked venom. In our study, it was observed that both method of venom extraction were different in terms of both quantity and quality which was confirmed by mass spectrometry.

Venom production was decreased gradually after 7–8 consecutive extractions for both species. Yaqoob et al. (2016) have reported lower quantity of venom after consecutive extractions from *A. finitimus* and *O. Odonturus*. Candido and Lucas (2004) have also observed that yield of venom was decreased from *Tityus serrulatus* after consecutive extractions. This might be due to the fact that venom gland of scorpions might have been destroyed due to the repetitive extractions but need to be confirmed.

In the feeding experiment, we observed that grasshopper nymphs and adults were the best diet for the scorpions to get higher yield of venom as compared to house crickets, moths, and house flies. We concluded that grasshoppers for the scorpions were better than other prey items because they are intrinsically more nutritious. We did not find any documented data addressing such change in venom quantity. Further investigation is required to establish this fact.

In our study, we found that activity of scorpions and venom milking was associated with temperature. During summer season, activity of scorpions and venom recovery was comparatively high than winter season; when both of these were decreased. Our results indicated that scorpions were more active at higher temperature in summer season. Many researchers also reported that activity of scorpions increases with increasing temperatures (Bridges et al. 1997, Ahsan and Tahir 2016). However; several publications are available which describe seasonal variation in activities of scorpions (Cristiane et al. 2010). Terblanche et al. (2007) studied the relationship between variation in metabolism of scorpion and temperature. Lighton et al. (2001) quantified the temperature dependence of metabolic rate of different scorpions. They observed that metabolic rate of scorpion is increased remarkably with temperature.

### Conclusion

It is concluded that electrical method of extraction is an efficient method to get good quality and higher quantity of venom. Furthermore, both scorpion species produce more quantity of venom when they had grasshopper prey as a food. In addition, activity of scorpions and venom milking was higher in warmer days.
